# A big data approach to metagenomics for all-food-sequencing

**DOI:** 10.1186/s12859-020-3429-6

**Published:** 2020-03-12

**Authors:** Robin Kobus, José M. Abuín, André Müller, Sören Lukas Hellmann, Juan C. Pichel, Tomás F. Pena, Andreas Hildebrandt, Thomas Hankeln, Bertil Schmidt

**Affiliations:** 1 Department of Computer Science, Johannes Gutenberg University, Mainz, 55099 Germany; 2 IPCA, Polytechnic Institute of Cávado and Ave, Barcelos, 4750-810 Portugal; 3 CiTIUS, Universidade de Santiago de Compostela, Santiago de Compostela, 15782 Spain; 4 Molecular Genetics and Genome Analysis, Institute of Organismal and Molecular Evolution, Johannes Gutenberg University, Mainz, 55099 Germany

**Keywords:** Next-generation sequencing, Metagenomics, Species identification, Eukaryotic genomes, Locality sensitive hashing, Big data

## Abstract

**Background:**

All-Food-Sequencing (AFS) is an untargeted metagenomic sequencing method that allows for the detection and quantification of food ingredients including animals, plants, and microbiota. While this approach avoids some of the shortcomings of targeted PCR-based methods, it requires the comparison of sequence reads to large collections of reference genomes. The steadily increasing amount of available reference genomes establishes the need for efficient big data approaches.

**Results:**

We introduce an alignment-free *k*-mer based method for detection and quantification of species composition in food and other complex biological matters. It is orders-of-magnitude faster than our previous alignment-based AFS pipeline. In comparison to the established tools CLARK, Kraken2, and Kraken2+Bracken it is superior in terms of false-positive rate and quantification accuracy. Furthermore, the usage of an efficient database partitioning scheme allows for the processing of massive collections of reference genomes with reduced memory requirements on a workstation (AFS-MetaCache) or on a Spark-based compute cluster (MetaCacheSpark).

**Conclusions:**

We present a fast yet accurate screening method for whole genome shotgun sequencing-based biosurveillance applications such as food testing. By relying on a big data approach it can scale efficiently towards large-scale collections of complex eukaryotic and bacterial reference genomes. AFS-MetaCache and MetaCacheSpark are suitable tools for broad-scale metagenomic screening applications. They are available at https://muellan.github.io/metacache/afs.html (C++ version for a workstation) and https://github.com/jmabuin/MetaCacheSpark (Spark version for big data clusters).

## Background

Monitoring of food ingredients is becoming an increasingly important task. Relevant issues include correct labeling, fraud detection, and assessment of health risks [1]. This motivates the need for analytical methods that allow for accurate determination and quantification of food ingredients ideally spanning all kingdoms of life including animals, plants, bacteria, fungi, and possibly even viruses.

Quantitative real-time polymerase chain reaction (qPCR) [2] and droplet digital PCR (ddPCR) [3] are DNA-based technologies for food control that are widely used in practice. Unfortunately, these methods are limited by the number of target species within a single assay and thus are not suitable for broad-scale species screening. Similar restrictions apply to approaches based on sequencing of species-specific DNA bar codes [4].

High-throughput sequencing of total metagenomic DNA from biological samples provides the possibility to screen for a wide range of species as it does not require any prior definition of possible target species. However, subsequent bioinformatic analysis of large amounts of sequence-reads is required to identify and quantify actual food components. Our All-Food-Seq (AFS) pipeline [5, 6] maps each sequenced read to a number of reference genomes and then determines species composition and relative quantities based on a read counting procedure. Evaluation based on simulated as well as real data has demonstrated that AFS can detect anticipated species in food products and achieve quantification accuracy comparable to qPCR.

However, the AFS pipeline relies on applying a read alignment tool (such as BWA [7–9], Bowtie2 [10], or CUSHAW [11]) for each considered reference genome. Thus, runtime scales linearly with the number of considered genomes. For example, the quantification of a typical short read dataset consisting of a few million reads using ten mammalian and avian reference genomes with the BWA-based AFS pipeline already requires several hours on a standard workstation (not including the time for index construction). For broader scale screening of many species a much larger amount of reference genomes would be required, making this approach unfeasible.

More recently, a number of innovative techniques for fast taxonomic labeling in the field of bacterial metagenomics have been proposed. Wood and Salzberg [12] demonstrated that a *k*-mer-based exact matching approach can achieve high read classification accuracy while being around three orders-of-magnitude faster than the alignment tool MegaBLAST. It relies on building a database of all substrings of length *k* of each considered (bacterial) reference genome. A read is classified by querying the database using each of its *k*-mers as query. If a query returns a match a counter for the corresponding reference genome(s) is incremented. Finally, a read is taxonomically labeled based on high-scoring counters. Recent benchmark studies [13, 14] demonstrated that *k*-mer based tools such as Kraken [12], Kraken2+Bracken [15], CLARK [16], and MetaCache [17] can produce superior read assignment accuracy compared to several other tools including MetaPhlAn [18], mOTU [19], QIIME [20], and Kaiju [21] for selected bacterial metagenomic datasets. While being accurate, the major drawback of the *k*-mer based approach is high main memory consumption and long database construction times. For typical bacterial reference genome sets the databases used by Kraken and CLARK already consume several hundreds of gigabytes in size. The significantly higher complexities of eukaryotic reference genomes relevant for monitoring food ingredients therefore make an extension of this method to food-monitoring challenging.

Here, we present a novel computational method for broad-scale detection and quantification of species composition in food and other complex biological matters. It is based on our recently introduced MetaCache [17] bacterial metagenomic read classification algorithm. We employ a big data technique called minhashing to subsample *k*-mers in an intelligent way, thereby reducing the amount of stored *k*-mers by an order-of-magnitude. In this paper we show how this method can be extended from the taxonomic labeling of bacterial reads to the detection and quantification of ingredients in food samples that can span various kingdoms of life. MetaCache is augmented with the ability to estimate the abundance of organisms at a selectable taxonomic level as well as the possibility to filter out target references based on sequence coverage. Furthermore, we combine the minhashing algorithm used by MetaCache with efficient partitioning schemes. This allows us to employ databases that index large collections of reference genomes efficiently in terms of both construction times and memory consumption. We present two partitioning schemes and provide corresponding implementations for standard workstations based on C++ (AFS-MetaCache) and for big data clusters based on Apache Spark (MetaCacheSpark). Both version can be used as substitutes for the alignment tools previously employed in the AFS pipeline.

Our experimental results using a number of sequenced calibrator sausages of known species composition show that AFS-MetaCache runs orders-of-magnitude faster than the alignment-based AFS pipeline while yielding similar results. Furthermore, AFS-MetaCache and MetaCacheSpark yield lower false-positive rates and higher quantification accuracy compared to Kraken2, Kraken2+Bracken, and CLARK. They also provide faster database construction times and competitive query speeds. Our database partitioning scheme allows the reduction of peak main memory consumption on a single workstation or a cluster node significantly and therefore enables scalability to growing genome collections.

## Implementation

### Approach

Many tools in metagenomics struggle to keep pace with the increasing amount of available reference genomes. We address this issue by aiming at species identification and quantification at a large scale by using a combination of two big data techniques.

Minhashing: We adopt *minhashing*– a locality sensitive hashing (LSH) based data subsampling technique. It has been successfully applied by search engines to detect near duplicate web pages [22] but has recently gained popularity in bioinformatics with example applications including genome assembly [23], sequence clustering [24], and privacy-preserving read mapping [25]. Mash Screen [26] also employs minhashing for metagenomic analysis. While it allows to identify genomes contained in a sample, Mash Screen is not able to classify individual reads or quantify abundances by itself. Partitioning: Because the RAM of a single workstation or a cluster node can become insufficient to hold a complete reference database, we employ a partitioning scheme to divide reference sequences into multiple chunks. The partitions can be queried successively on a single workstation or among multiple worker nodes of a distributed compute cluster. In order to support these two types of compute resources we have developed (*i*) AFS-MetaCache: a C++ version for individual workstations, and (*ii*) MetaCacheSpark: a distributed version based on the big data analytics engine Apache Spark [27] for compute clusters.

### Database construction

Consider a collection *G* of *m* genomic sequences (reference genomes). Each reference genome is divided into windows of size *l* which overlap by *k*−1 base-pairs. Typically, *l* is of similar size to the anticipated read length (e.g. *l*=128 for Illumina data as default). For each window a *sketch* is calculated using minhashing. A sketch consists of the *s* smallest *k*-mers (in strand-neutral canonical representation) contained in the window with respect to an applied hash function *h*_1_. Thus, the sketching procedure selects only a subset of *k*-mers to be inserted into the database used for similarity computation. Assuming unique *k*-mers, the subsampling factor can be determined as $S = \frac {l-k+1}{s}$; i.e. for typical values such as *s*=8, *k*=16, and *l*=128 this corresponds to a data reduction by over an order-of-magnitude (*S*=14.125). Besides providing data reduction, minhashing also exhibits a desirable mathematical property when comparing two sketches: The relative intersection ratio between two sketched windows approximates the true Jaccard index evaluated on the whole *k*-mer space [22].

The hash table (database) for a given collection of reference genomes is constructed using open addressing. The entries of the hash table consist of key-target-list pairs. An associated hash function *h*_2_ maps *k*-mers to slots in the hash table. If an identified slot is empty or occupied with the same *k*-mer, the corresponding *k*-mer is inserted as key and the corresponding location (genome ID, window ID) is appended to the target-list. If the slot is occupied by a different *k*-mer quadratic probing is used to iterate to the next slot. Target lists have a pre-defined maximum length. If the maximum length is reached, the corresponding *k*-mer is considered uninformative and deleted from the hash table at the end of the construction.

**Fig. 1:**
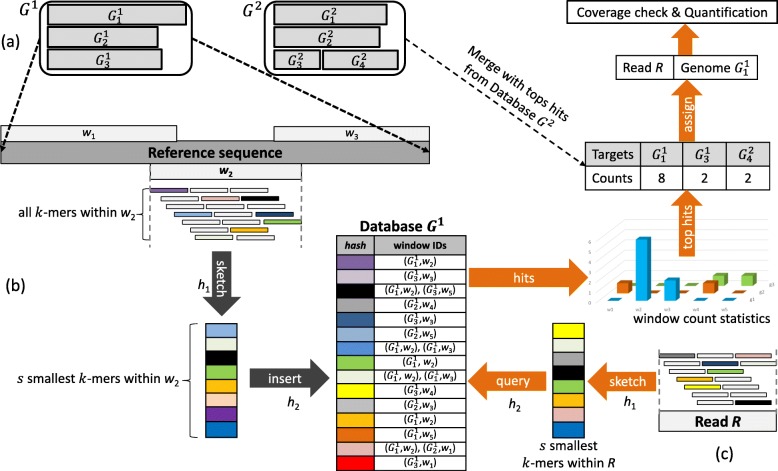
Workflow: (**a**) Partitioning: reference sequences are divided into the sets *G*^1^ and *G*^2^. Each reference is further partitioned into slightly overlapping windows *w*_*i*_. (**b**) Database construction: the *s* smallest *k*-mers of each window are computed and inserted into the database. (**c**) Classification: a database is queried with the *s* smallest *k*-mers of a read. The returned hits are used to count the number of hits within each window. Target reference genomes are identified by high scores in the window count statistics. In case of several partitions, the top hits from querying each database need to be merged in order to assign a read to a reference genome. After all reads have been processed, coverage check and quantification are performed

In the big data scenario we need to consider cases where the database is too large to fit into the RAM of a single workstation or a cluster node. Hence, it needs to be split into multiple parts which can be queried successively or distributed among multiple worker nodes of a cluster. Partitioning divides the collection of reference genomes *G* of total base-pair length *M* into disjoint buckets $G = \bigcup _{i=1}^{n}G^{i}$ of roughly equal size; i.e. $G^{i} = \{ G^{i}_{1}, \ldots, G^{i}_{n_{i}}\}$ where $ N_{i} = \sum _{j=1}^{n_{i}} \left | G^{i}_{j} \right | \approx M/n$. The partition size *N*_*i*_ can be chosen depending on the available main memory resources and the subsampling factor *S*. For each partition *G*^*i*^ a separate hash table (database) is constructed by the aforementioned method. Our partitioning scheme is illustrated in Fig. 1(a) and database construction in Fig. 1(b).

#### Single workstation

AFS-MetaCache constructs a separate database for each partition of reference sequences *G*^*i*^ and stores it as a database file on disk. We also allow to add sequences to previously constructed databases. This makes it easy to modify the set of reference genomes by either swapping out database partitions or including more sequences.

#### Spark

**Fig. 2:**
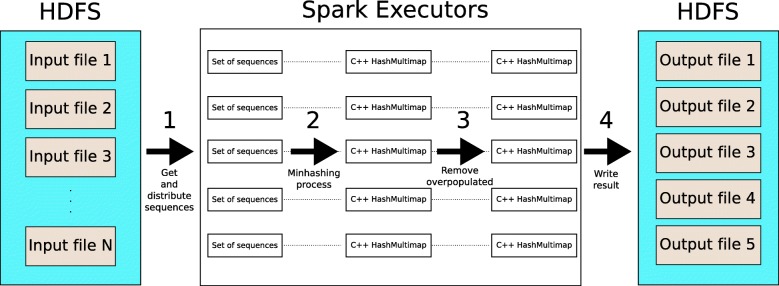
Database construction algorithm used by MetaCacheSpark

Apache Spark is a distributed memory computing engine [27]. It is able to process a large quantity of input data in parallel thanks to the combination of the Hadoop Distributed File System (HDFS) and Resilient Distributed Datasets (RDDs). These two features are used by MetaCacheSpark. Our algorithm consists of four phases that are illustrated in Fig. 2.

1. Reference genome sequences are loaded from HDFS and distributed proportionally among the Spark executors. In this way, each executor will contain a different subset of sequences to work with.
2. With these sequences loaded into memory, the Spark executors perform the described minhashing algorithm. Results are stored in a executor-local C++ hash table, similar to the one used by AFS-MetaCache.
3. We apply a map-reduce operation where the map operator receives the number of items belonging to the same key in each executor, and the reduction phase sums up the number of items calculating a global count. If the global item count per key exceeds a given threshold (by default 254), the corresponding items are deleted from all the executor-local hash tables.
4. Each hash table is written to a database file stored in HDFS.

At the end of the process, each executor will contain one, and only one, hash table. Note that a key can be present in several hash tables. However, items belonging to the same target ID (i.e., to the same reference sequence) will be present only in one hash table (this is important for the subsequent read assignment phase).

Furthermore, both versions have a pre-processing phase prior to database construction that builds a taxonomic tree of the considered reference genomes.

### Individual read assignment

In order to assign reads to reference genome(s) minhashing is applied to any given read *R* in the same way as to a reference genome window using the hash function *h*_1_. The produced sketch is used to query a loaded hash table using the hash function *h*_2_. Each query returns a (possibly empty) target list. The target lists are merged into a sparse two-dimensional data structure (called *window count statistic*) by accumulating identical (genome ID, window ID) pairs. High values in the window count statistic indicate a match of the read in the corresponding genome. The counts are sorted in descending order and the targets with the highest counts are considered in order to classify a read. This process is illustrated in Fig. 1(c).

However, a match of a paired-end (or even a single-end) read typically corresponds to a region in the genome that overlaps the borders of two or more windows in this genome. Thus, we accumulate the counters spanning a contiguous range of several neighboring windows to find the ranges with maximum hit counts. The considered read is assigned to the genome containing the best final count if it is significantly higher than the second best. If the count difference is small, the read is assigned to the lowest common ancestor (LCA) of multiple candidate genomes which are in a similar count range using the provided taxonomic tree.

#### Single workstation

AFS-MetaCache reads the database partitions from disk and queries them with the set of reads in succession. Subsequently, the individual results are merged to determine the final classification for each read. We further support multi-threading by processing chunks of reads independently in order to exploit multiple CPU cores.

#### Spark

**Fig. 3:**
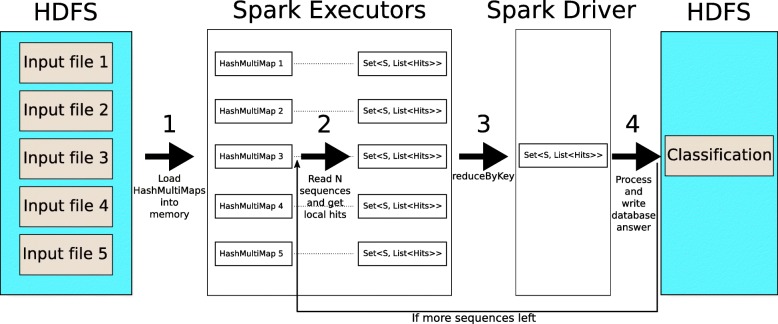
Individual read classification algorithm used by the MetaCacheSpark

Two inputs are needed: the database files created in the build phase and the input reads to be processed. The MetaCacheSpark algorithm consists of four steps (see Fig. 3):

1. Each hash table is loaded into the main memory of one executor. Furthermore, the taxonomy is loaded only in the Spark driver.
2. All executors read a block of *N* input reads to be processed from HDFS. Note that every executor needs to read all of them since the hash table is distributed. While reading the input sequences, each executor queries its local hash table to compute the (local) classification candidates with their corresponding hits. This process returns a set of key-value pairs, where the key is the ID of the read being processed, and the value is a list of possible candidates with their corresponding hit counts.
3. The next step is a reduction phase. Here, partial results from each executor are grouped using read IDs as keys. The driver then collects the *N* results and performs the assignment of reads to reference genomes (classification). This step uses the Spark function *reduceByKey()*, and it requires a *shuffle*.
4. Classification results from the previous step are written to the output file in HDFS. The algorithm goes back to Step 2 to process the next chunk of reads.

It is also important to note that:

- There is a guarantee that items belonging to the same reference sequence during the build phase are present in the same local hash table. Otherwise, calculating the hits in Step 2 would involve a distributed operation (such as *groupByKey()*) that would cause severe performance degradation.
- To gain speed, we further support multi-threading. Each thread processes a different set of input reads by means of a map-reduce job that corresponds to Steps 3 and 4.
- The reduction generates a lot of traffic over the network and requires an expensive shuffle operation. In order to reduce the associated communication overhead, we have introduced an optional parameter (*H*) that is used to discard all candidates in Step 2 and Step 3 with less than *H* hits. However, if this parameter is used, results can be slightly different compared to the single workstation version.

### Coverage filter

False positive read assignments can be caused by shared regions of DNA among multiple reference genomes [28]. We use coverage information to detect some of these cases as follows.

Before assigning reads to classification targets we can filter the list of candidate genomes identified during the read assignment phase by checking the coverage per genome as follows. We analyze which windows of a target genome are covered by reads from the dataset. If the percentage of covered windows of a genome is much lower compared to other genomes, it is likely to be a false positive and will be deleted from the list of possible target genomes. In fact we delete a quantile (e.g. 10%) of the target genomes with the lowest coverage. The reads are then classified with respect to the remaining genomes.

Note that this strategy is only applicable if the number of reads is large enough to cover significant parts of the genomes. In our experience it proofed especially efficient in case of bacterial genomes which are orders of magnitudes smaller than animal or plant genomes.

### Quantification

In addition to the per-read classification we are able to estimate the abundances of organisms contained in a dataset at a specific taxonomical rank. For each taxon which occurs in the dataset we count the number of reads assigned to it. We then build a taxonomic tree containing all found taxa.

Taxa on lower levels than the requested taxonomic rank are pruned and their read counts are added to their respective parents, while reads from taxa on higher levels are distributed among their children in proportion to the weights of the sub-trees rooted at each child. After the redistribution the estimated number of reads and abundance percentages are returned as outputs.

## Results

### Datasets

In order to measure performance and accuracy of our approach in comparison to other metagenomic tools, we have created databases of varying size containing different organisms. Food-related genomes (selection of main ingredients) used for database construction are listed in Table 1 while the considered bacteria, viruses, and archaea from NCBI RefSeq (Release 90) are summarized in Table 2. The created databases with their included reference genomes are described in Table 3.

**Table 1:** Food-related reference genomes used for database construction

**Item**	**Name**	**ID**	**Size on disk**
1	Sus scrofa (pig)	GCF_000003025.6	2.4GB
2	Equus caballus (horse)	GCF_002863925.1	2.4GB
3	Meleagris gallopavo (turkey)	GCF_000146605.2	1.2GB
4	Mus musculus (house mouse)	GCF_000001635.26	2.7GB
5	Gallus gallus (chicken)	GCF_000002315.5	1.1GB
6	Ovis aries (sheep)	GCF_000298735.2	2.5GB
7	Rattus norvegicus (Norway rat)	GCF_000001895.5	2.8GB
8	Bos taurus (cattle)	GCF_002263795.1	2.6GB
9	Bubalus bubalis (water buffalo)	GCF_003121395.1	2.6GB
10	Cervus elaphus hippelaphus (red deer)	GCA_002197005.1	3.3GB
11	Capreolus capreolus (Western roe deer)	GCA_000751575.1	3.0GB
12	Struthio camelus australis (African ostrich)	GCA_000698965.1	1.2GB
13	Anas platyrhynchos (mallard)	GCF_003850225.1	1.1GB
14	Capra hircus (goat)	GCF_001704415.1	2.8GB
15	Oryctolagus cuniculus (rabbit)	GCF_000003625.3	2.6GB
16	Cavia aperea (Brazilian guinea pig)	GCA_000688575.1	2.6GB
17	Camelus ferus (Wild Bactrian camel)	GCF_000311805.1	1.9GB
18	Canis lupus familiaris (dog)	GCF_000002285.3	2.3GB
19	Felis catus (domestic cat)	GCF_000181335.3	2.4GB
20	Homo sapiens (human)	GCF_000001405.38	3.1GB
21	Equus asinus (ass)	GCA_001305755.1	2.3GB
22	Rangifer tarandus (reindeer)	GCA_004026565.1	2.9GB
23	Phasianus colchicus (Ring-necked pheasant)	GCA_004143745.1	987MB
24	Glycine max (soybean)	GCF_000004515.5	946MB
25	Zea mays (maize)	GCF_000005005.2	2.1GB
26	Triticum aestivum (bread wheat)	GCA_900519105.1	14.0GB
27	Secale cereale (rye)	GCA_900079665.1	1.8GB
28	Hordeum vulgare (barley)	GCA_004114815.1	3.8GB
29	Oryza sativa Japonica Group (Japanese rice)	GCF_001433935.1	362MB
30	Arachis hypogaea (peanut)	GCF_003086295.1	2.4GB
31	Saccharomyces cerevisiae S288C (baker’s yeast)	GCA_000146045.2	12MB
**Total**			**74GB**

**Table 2:** Reference genomes from NCBI RefSeq (Release 90) used for database construction

**Organism**	**Number of references**	**Size on disk**
Bacteria	10838	41.0GB
Viral	7857	269MB
Archaea	269	656MB
**Total**	**18964**	**41.9GB**

**Table 3:** Data sets used for database construction

**Name**	**Number of species**	**Size on disk**
**AFS10**	Animal genomes from 1 to 10	22.3GB
**AFS20**	Animal genomes from 1 to 20	45.8GB
**AFS20RS90**	Animal genomes from 1 to 20 plus NCBI RefSeq (Release 90)	87.5GB
**AFS31**	Animal genomes from 1 to 31	76.8GB
**AFS31RS90**	Animal genomes from 1 to 31 plus NCBI RefSeq (Release 90)	118.5GB

We use ten short read datasets sequenced from calibrator sausage samples containing admixtures of a set of food relevant ingredients (chicken, turkey, pork, beef, horse, sheep) on an Illumina HiSeq machine (downloaded from ENA project ID PRJNA271645 (Kal_D and KAL_D) and PRJEB34001 (all other data)). Table 4 shows the read datasets together with the corresponding percentage of meat components used during preparation. The samples comprise meat proportions ranging from 0.5% to 80% and can be subdivided into two categories: Kal A-E consist only of mammalian meat, while KLyo A-D represent Lyoner-like sausages containing poultry in addition to mammals [29, 30]. The dataset KAL_D is identical to Kal_D but sequenced with higher coverage.

**Table 4:** Calibrator sausage datasets and their meat composition

**Name**	**#Reads (paired-end)**	**Cattle**	**Sheep**	**Pig**	**Horse**	**Chicken**	**Turkey**
KLyo_A	401K	14.0%	0.0%	80.0%	0.0%	0.5%	5.5%
KLyo_B	302K	36.0%	0.0%	58.0%	0.0%	2.0%	4.0%
KLyo_C	507K	58.0%	0.0%	36.0%	0.0%	4.0%	2.0%
KLyo_D	417K	80.0%	0.0%	14.0%	0.0%	5.5%	0.5%
Kal_A	830K	1.0%	9.0%	35.0%	55.0%	0.0%	0.0%
Kal_B	977K	9.0%	1.0%	55.0%	35.0%	0.0%	0.0%
Kal_C	404K	25.0%	25.0%	25.0%	25.0%	0.0%	0.0%
Kal_D	403K	35.0%	55.0%	9.0%	1.0%	0.0%	0.0%
Kal_E	289K	55.0%	35.0%	1.0%	9.0%	0.0%	0.0%
KAL_D	26,114K	35.0%	55.0%	9.0%	1.0%	0.0%	0.0%

### Quantification accuracy

Tables 5 and 6 show the quantification results returned by the tested tools (AFS-MetaCache (v.0.5.3), MetaCacheSpark, CLARK (v.1.2.6), Kraken2 (v.2.0.7-beta), and Kraken2 with subsequent abundance estimation by Bracken v.2.0.0 – all executed with default parameters) using AFS20 as reference database. Besides showing the quantification for each included meat component, we also show the (false positive) results for water buffalo (closely related to cattle) and goat (closely related to sheep). In addition, we provide the sum of all false positive (*Σ* FP) read classifications over all of the detected reference genomes that were not included in the sample. In addition, the sum of the deviations of the measured proportions to the real sausage composition (*Σ* Dev) as well as the averages over all tested datasets are shown.

**Table 5:** Quantification results for the Klyo samples using the reference dataset AFS20 and the average result for AFS31RS90

**Dataset**	**Classifier**	**Cattle**	**Pig**	**W.Buf.**	**Goat**	**Chicken**	**Turkey**	***Σ* FP**	***Σ* Dev**
KLyo_A	Expected	14.0%	80.0%	0.00%	0.00%	0.50%	5.50%		
	AFS-MC	16.6%	71.5%	0.04%	0.02%	0.60%	4.64%	**0.28**%	**12.39**%
	MCSpark	16.9%	71.2%	0.04%	0.02%	0.60%	4.64%	0.32%	12.99%
	CLARK	16.4%	70.4%	0.20%	0.09%	0.62%	4.61%	0.51%	13.55%
	Kraken2	15.9%	70.0%	0.27%	0.11%	0.65%	4.59%	0.87%	13.82%
	K2+Brack	17.6%	70.3%	0.30%	0.14%	0.66%	4.63%	0.97%	15.33%
KLyo_B	Expected	36.0%	58.0%	0.00%	0.00%	2.00%	4.00%		
	AFS-MC	37.6%	51.0%	0.12%	0.04%	2.05%	2.99%	**0.50**%	10.16%
	MCSpark	37.9%	50.5%	0.12%	0.04%	2.06%	3.02%	0.60%	11.11%
	CLARK	35.9%	50.4%	0.47%	0.19%	2.10%	3.01%	1.03%	**9.84**%
	Kraken2	34.5%	49.9%	0.68%	0.24%	2.12%	2.99%	1.57%	12.11%
	K2+Brack	39.1%	50.2%	0.32%	0.78%	2.15%	3.02%	1.84%	13.93%
KLyo_C	Expected	58.0%	36.0%	0.00%	0.00%	4.00%	2.00%		
	AFS-MC	57.7%	27.1%	0.16%	0.06%	3.56%	1.16%	**0.95**%	**11.47**%
	MCSpark	57.7%	26.9%	0.16%	0.06%	3.63%	1.18%	0.95%	11.48%
	CLARK	54.1%	25.9%	0.69%	0.29%	3.58%	1.16%	1.88%	17.11%
	Kraken2	52.2%	25.7%	0.95%	0.36%	3.57%	1.17%	2.58%	19.94%
	K2+Brack	58.6%	25.8%	1.07%	0.46%	3.60%	1.18%	2.89%	14.90%
KLyo_D	Expected	80.0%	14.0%	0.00%	0.00%	5.50%	0.50%		
	AFS-MC	74.7%	10.9%	0.23%	0.08%	4.66%	0.33%	**0.93**%	10.27%
	MCSpark	74.7%	10.8%	0.23%	0.08%	4.69%	0.33%	1.09%	10.58%
	CLARK	70.8%	10.8%	0.94%	0.39%	4.73%	0.35%	1.94%	15.27%
	Kraken2	68.0%	10.7%	1.26%	0.48%	4.70%	0.36%	2.42%	18.62%
	K2+Brack	77.6%	10.8%	1.45%	0.62%	4.76%	0.36%	2.87%	**9.35**%
Average	AFS-MC			**0.14**%	**0.05**%			**0.67**%	**11.07**%
	MCSpark			**0.14**%	**0.05**%			0.74%	11.54%
	CLARK			0.58%	0.24%			1.34%	13.94%
	Kraken2			0.79%	0.30%			1.86%	16.12%
	K2+Brack			0.71%	0.50%			2.14%	13.38%
AFS31RS90	AFS-MC							**0.58**%	**13.97**%
Average	MCSpark							0.59%	14.08%

AFS-MC: AFS-MetaCache, MC-Spark: MetaCacheSpark, K2+Brack: Kraken2 with subsequent Bracken, W.Buf: Water Buffalo, *Σ* FP: Sum of all false positive read classifications, *Σ* Dev: Sum of absolute deviations to the given meat composition (best results for each dataset in bold)

**Table 6:** Quantification results for the Kal samples using the reference dataset AFS20 and the average result for AFS31RS90

**Dataset**	**Classifier**	**Cattle**	**Sheep**	**Pig**	**Horse**	**W.Buf.**	**Goat**	***Σ* FP**	***Σ* Dev**
Kal_A	Expected	1.00%	9.0%	35.0%	55.0%	0.00%	0.00%		
	AFS-MC	1.25%	11.0%	30.5%	54.1%	0.01%	0.29%	**0.42**%	8.13%
	MCSpark	1.27%	11.1%	30.3%	54.1%	0.01%	0.29%	0.45%	8.42%
	CLARK	1.29%	9.1%	31.1%	54.0%	0.09%	0.89%	1.15%	**6.43**%
	Kraken2	1.23%	8.7%	30.9%	53.9%	0.08%	0.96%	1.31%	6.99%
	K2+Brack	1.43%	10.3%	31.0%	54.0%	0.10%	1.12%	1.53%	8.24%
Kal_B	Expected	9.0%	1.00%	55.0%	35.0%	0.00%	0.00%		
	AFS-MC	10.5%	1.42%	49.3%	35.6%	0.03%	0.06%	**0.27**%	8.43%
	MCSpark	10.6%	1.42%	49.1%	35.7%	0.03%	0.06%	0.30%	8.92%
	CLARK	10.3%	1.26%	50.0%	35.8%	0.17%	0.18%	0.56%	**7.85**%
	Kraken2	10.0%	1.21%	49.6%	35.7%	0.20%	0.20%	1.03%	8.40%
	K2+Brack	11.0%	1.40%	35.8%	49.7%	0.22%	0.23%	1.09%	9.60%
Kal_C	Expected	25.0%	25.0%	25.0%	25.0%	0.00%	0.00%		
	AFS-MC	23.3%	29.6%	19.2%	23.0%	0.06%	0.73%	**1.08**%	15.28%
	MCSpark	23.5%	29.6%	19.0%	22.9%	0.06%	0.73%	1.18%	15.32%
	CLARK	23.4%	25.6%	19.4%	23.2%	0.45%	2.56%	3.38%	**12.98**%
	Kraken2	22.7%	24.7%	19.4%	23.1%	0.49%	2.69%	3.48%	13.65%
	K2+Brack	24.8%	27.8%	19.4%	23.2%	0.54%	3.02%	3.89%	14.35%
Kal_D	Expected	35.0%	55.0%	9.00%	1.00%	0.00%	0.00%		
	AFS-MC	32.9%	51.5%	7.14%	1.14%	0.09%	1.50%	**2.07**%	**9.62**%
	MCSpark	33.2%	51.2%	7.03%	1.13%	0.09%	1.49%	2.23%	9.91%
	CLARK	32.8%	43.1%	7.31%	1.16%	0.72%	4.40%	5.69%	21.61%
	Kraken2	31.6%	41.3%	7.26%	1.16%	0.79%	4.62%	5.77%	24.75%
	K2+Brack	35.8%	48.4%	7.28%	1.16%	0.89%	5.40%	6.70%	15.96%
Kal_E	Expected	55.0%	35.0%	1.00%	9.00%	0.00%	0.00%		
	AFS-MC	50.4%	33.7%	0.99%	7.80%	0.12%	0.96%	**1.52**%	**8.55**%
	MCSpark	50.7%	33.4%	0.97%	7.73%	0.12%	0.95%	1.66%	8.82%
	CLARK	50.7%	28.7%	1.02%	7.81%	0.84%	3.07%	4.43%	16.26%
	Kraken2	49.2%	27.6%	1.00%	7.80%	0.99%	3.28%	4.58%	18.96%
	K2+Brack	54.1%	31.4%	1.00%	7.81%	1.10%	3.71%	5.15%	10.86%
KAL_D	Expected	35.0%	55.0%	9.00%	1.00%	0.00%	0.00%		
	AFS-MC	30.3%	49.6%	7.27%	1.16%	0.08%	1.25%	1.38%	**13.36**%
	MCSpark	30.4%	49.5%	7.25%	1.16%	0.08%	1.26%	**1.36**%	**13.36**%
	CLARK	30.8%	43.3%	7.51%	1.20%	0.86%	4.57%	6.30%	23.85%
	Kraken2	29.6%	41.3%	7.47%	1.19%	0.95%	4.98%	7.03%	27.86%
	K2+Brack	33.5%	48.7%	7.58%	1.19%	1.08%	5.84%	8.07%	17.44%
Average	AFS-MC					**0.07**%	**0.80**%	**1.12**%	**10.56**%
	MCSpark					**0.07**%	**0.80**%	1.20%	10.79%
	CLARK					0.51%	2.61%	3.59%	14.83%
	Kraken2					0.58%	2.79%	3.87%	16.77%
	K2+Brack					0.66%	3.22%	4.41%	12.74%
AFS31RS90	AFS-MC							**1.84**%	**13.38**%
Average	MCSpark							**1.84**%	13.63%

AFS-MC: AFS-MetaCache, MC-Spark: MetaCacheSpark, K2+Brack: Kraken2 with subsequent Bracken, W.Buf: Water Buffalo, *Σ* FP: Sum of all false positive read classifications, *Σ* Dev: Sum of absolute deviations to the given meat composition (best results for each dataset in bold)

In terms of sensitivity, all methods are able to detect the included meat components. In addition, several tools detect false positive signals; e.g., Kraken2+Bracken detects over 1% of water buffalo in KLyo_C and KLyo_D and over 3% of goat in Kal_C, Kal_D, and Kal_E. False positive quantities in these cases correlate with the amount of beef and the amount of sheep present in the respective sample. Overall, AFS-MetaCache achieves the lowest FP-rates for each tested dataset with an average FP-sum per sample of only 0.67% for the Klyo samples and 1.12% for the Kal samples. This is much lower compared to CLARK (1.34% for Klyo, 3.59% for Kal), Kraken2 (1.86% for Klyo, 3.87% for Kal), and Kraken2+Bracken (2.14% for Klyo, 4.41% for Kal). The relative differences become even more significant when looking at some of the individual FP signals. In the Klyo samples (Table 5) AFS-MetaCache only detects negligible amounts of goat (0.05% on average) and water buffalo (0.14%), while the amounts detected by CLARK, Kraken2, and Kraken2+Bracken are higher by factors of 4.2 and 4.8, 5.6 and 6.0, and 5.1 and 10.0, respectively. Similar results can be observed for the Kal samples (Table 6): AFS-MetaCache only detects 0.07% of water buffalo meat on average and 0.80% of goat meat on average, while the amounts detected by CLARK, Kraken2, and Kraken2+Bracken are higher by factors of 7.3 and 3.3, 8.3 and 3.5, and 9.4 and 4.0, respectively.

In terms of deviation from the expected foodstuff ingredients, AFS-MetaCache shows the lowest average of the sums of absolute differences for both Klyo (11.07%) samples and Kal samples (10.56%). Kraken2+Bracken (13.38% and 12.74%) has smaller deviations on average than Kraken2 alone (16.12% and 16.77%), showing that quantification after read assignment is beneficial.

As can be seen in Tables 5 and 6 there are small differences between the results of AFS-MetaCache and MetaCacheSpark. They are caused by the constraint list of target genomes with highest scores (tophits) of MetaCacheSpark and by the different ordering of targets with the same score. The differences could be reduced by increasing the tophits list size, but we decided for a smaller list in favor of faster querying speeds.

When scanning the calibrator sausage read datasets with AFS-MetaCache using the bigger AFS31 and AFS31RS90 databases, we can make the following observations: (1) More *k*-mers are removed from the hash table due to overflowing target lists. Therefore, the number of classified reads is reduced and total deviation increases slightly. (2) Additional false positive targets are introduced, but the total number of false positives is reduced for the Klyo datasets (excluding bacteria).

A benefit of screening for microbiota and eukaryotic foodstuff species at the same time is a lower false positive rate. Usually reads of a dataset are queried against either one or the other and only the remaining unclassified reads are investigated further. This can lead to false assumptions about the data. In our experiments some reads are falsely classified as Triticum aestivum (bread wheat) when using the AFS31 database. With the AFS31RS90 database, however, those reads are identified as bacterial or unspecific (classified as the lowest common ancestor of bread wheat and bacteria).

### Runtime and memory consumption for non-Partitioned databases

Runtime and memory consumption where the whole database can fit into the available main memory are measured on a system with a dual Xeon E5-2630v4 (2.2 GHz, 2×10 cores) CPU with 512 GB of DDR4 RAM. We have compared the speed and the peak memory consumption during database construction and classification of the default versions of AFS-MetaCache (v.0.5.3), CLARK (v.1.2.6), Kraken2 (v2.0.7-beta), and Kraken2 with subsequent abundance estimation by Bracken v.2.0.0 (Kraken2+Bracken) using 40 threads. Table 7 shows the results for the reference genome datasets listed in Table 3 and the KAL_D read dataset (26 million paired-end reads of length 101 bp) for classification. Note, that the time to load the databases is excluded when measuring query speed for all programs to make the results independent of dataset size.

**Table 7:** Runtimes and peak memory consumption for non-partitioned database construction (build) and querying for different data sets on a workstation with 512 GB RAM

**Data set**		**AFS-MetaCache**	**CLARK**	**Kraken2**	**Kraken2+Bracken**
AFS20	Build time	**1h 11m**	15h 37m	1h 27m	5h 32m
	Build memory	**64 GB**	428 GB	69 GB	147 GB
	Query time	136 s	93 s	**37 s**	111 s
	Query speed	11.5 MR/m	16.9 MR/m	**43.2 MR/m**	14.2 MR/m
	Query memory	**50 GB**	152 GB	54 GB	54 GB
AFS31	Build time	**1h 47m**	-	3h 19min	11h 41min
	Build memory	**91 GB**	-	107 GB	296 GB
	Query time	175 s	-	**44 s**	58 s
	Query speed	8.9 MR/m	-	**35.9 MR/m**	27.0 MR/m
	Query memory	78 GB	-	**72 GB**	**72 GB**
AFS20RS90	Build time	**1h 42m**	-	2h 58m	8h 53m
	Build memory	110 GB	-	**94 GB**	168 GB
	Query time	180 s	-	**43 s**	117 s
	Query speed	8.7 MR/m	-	**37.0 MR/m**	13.5 MR/m
	Query memory	94 GB	-	**79 GB**	**79 GB**
AFS31RS90	Build time	**3h 10m**	-	5h 55min	17h 44min
	Build memory	135 GB	-	**134 GB**	329 GB
	Query time	217 s	-	**49 s**	61 s
	Query speed	7.2 MR/m	-	**32.1 MR/m**	25.7 MR/m
	Query memory	117 GB	-	**97 GB**	**97 GB**

Query speeds are measured for the KAL_D dataset in terms of million reads per minute (MR/m). For the cases with “-” the corresponding program exceeds the main memory capacity of 512 GB. Fastest runtimes and lowest memory consumption for each dataset are indicated in bold

AFS-MetaCache is fastest for database construction for all tested data sets. Furthermore, it requires least memory for constructing the database for AFS20 and AFS31, but requires slightly more memory than Kraken2 for AFS20RS90 and AFS31RS90.

Kraken2 is fastest in terms of query (classification) speed. If Kraken2 is executed with subsequent quantification by Bracken, corresponding runtimes increase. Even though query speeds of MetaCache-AFS are slowest, corresponding execution times are still competitive (only around three minutes for the largest data set (KAL_D)).

For common data set sizes in food control applications runtimes for database construction (a few hours) are typically much higher than for the classification stage (a few minutes). Since the amount of relevant reference genomes is increasing rapidly corresponding databases have to be constructed or extended frequently. Thus, fast built times are of high importance. Besides having the fastest database construction time, AFS-MetaCache is also the only tool that supports the functionality of extending an existing database.

### Runtime and memory consumption for partitioned databases

In this subsection we evaluate the ability of AFS-MetaCache and MetaCacheSpark to reduce the consumed main memory by partitioning the database into smaller chunks. AFS-MetaCache is again evaluated on a workstation with a dual Xeon E5-2630v4 CPU and 512 GB of DDR4 RAM. MetaCacheSpark has been tested on a big data cluster composed of 12 Dell EMC PowerEdge R730 servers, each one with a dual Xeon E5-2630v4 (2.2GHz 10 cores) CPU with 384 GB RAM and 32 TB HDDs running Java version Openjdk 1.8.0_201, gcc 7.3.1, Spark 2.2.0, and Hadoop 2.7.3.

Table 8 shows the speed and memory consumption of AFS-MetaCache and MetaCacheSpark for partitioned database construction and querying using the AFS31RS90 reference genome dataset and the KAL_D dataset. Using four partitions, AFS-MetaCache can reduce the main memory consumption from 135 GB to only 52 GB while the construction time only slightly increases from 3h 10m to 3h 45m. In addition, memory consumption for classification is reduced from 117 GB to 39 GB. However, the corresponding query speed decreases from 7.2 MR/m to 2.5 MR/m since the partitions have to be queried by all reads in succession and the individual results need to be merged.

**Table 8:** Partitioned build time and query speed for AFS31RS90 database

**Tool**	**Build time**	**Max. Memory**	**Query Speed**	**Max. Memory**
AFS-MetaCache (1 part.)	3h 10min	135 GB	7.2 MR/m	117 GB
AFS-MetaCache (2 part.)	3h 04min	82 GB	3.1 MR/m	70 GB
AFS-MetaCache (4 part.)	3h 45min	52 GB	2.5 MR/m	39 GB
MetaCacheSpark (8Ex-32Th)	2h 57min	175 GB	4.3 MR/m	76 GB
MetaCacheSpark (16Ex-16Th)	1h 57min	100 GB	3.4 MR/m	48 GB
MetaCacheSpark (32Ex-8Th)	1h 25min	69 GB	2.2 MR/m	37 GB
MetaCacheSpark (64Ex-4Th)	1h 03min	45 GB	1.4 MR/m	29 GB

Query speed measured for dataset KAL_D in million reads per minute (MR/m). For MetaCacheSpark, the number of executors and threads per executor are indicated

The results show that memory requirements per node and build time for MetaCacheSpark both decrease when increasing the number of executors. As the number of executors increases, the benefits of using the Spark version are revealed. For 64 executors the AFS31RS90 database can be built in around one hour using 45 GB of memory per node. This is 3 ×, 3.6 ×, 5.6 ×, 16.9 × faster than AFS-MetaCache, 4-partitioned AFS-MetaCache, Kraken2 and Kraken2+Bracken, respectively. Important reductions in the memory consumed per node can also be observed.

MetaCacheSpark consumes less memory in the classification phase than in the build phase. Some additional memory is required to store query hits. However, this memory can be re-used with each batch of sequences being classified. As a trade-off to fast build time and low memory consumption per node, the query speeds of MetaCacheSpark are lower compared to non-partitioned AFS-MetaCache. This can be explained by the necessity to perform a costly shuffle operation for the reduce-by-key function. Its cost increases with the number of executors as can be seen in Table 8: query speed reduces from 4.2 MR/m with 8 executors to 1.4 MR/m with 64. Nevertheless, runtimes are still acceptable in application scenarios where relevant read datasets are small compared to the utilized databases.

### Comparison to previous aFS pipeline

To compare AFS-MetaCache to our previous alignment-based AFS pipeline the same dual-socket workstation as before is used. Runtimes and memory consumption of both approaches are shown in Table 9. For the small genome dataset AFS10 the previous AFS pipeline already takes several hours to construct the index. Querying of the KAL_D dataset takes even more than 10 hours. For bigger numbers of reference genomes this approach becomes unfeasible because the runtime scales linearly with the number reference genomes. On the other hand, AFS-MetaCache takes less than an hour for database construction of AFS10 while the query speed improves by more than two orders of magnitude. As shown before even larger databases like AFS31 can be built by AFS-MetaCache in just a few hours and query speed drops by less than a factor of two.

**Table 9:** Runtimes and peak memory consumption for database construction (build) and querying for AFS10

**Data set**		**AFS-MetaCache**	**AFS-previous**
AFS10	Build time	**47m**	7h 0m
	Build memory	35 GB	**5GB**
	Query speed	**17.1 MR/m**	0.04 MR/m
	Query memory	30 GB	**6GB**

Query speeds are measured for the KAL_D dataset in terms of million reads per minute (MR/m)

The average quantification results for the Klyo and Kal samples produced by AFS-MetaCache and the previous AFS pipeline are shown in Table 10. The *k*-mer based AFS-MetaCache is able to match quantification accuracy of the previous alignment-based pipeline for the KLyo datasets. The average deviation to the meat components is even lower for AFS-MetaCache. For the Kal datasets AFS-MetaCache reduces the false positive rate while the average deviation increases slightly. However, it is still possible to identify the correct components with the benefit of less false positives.

**Table 10:** Average quantification results for the Klyo and Kal samples using the reference dataset AFS10

**Dataset**	**Classifier**	***Σ* FP**	***Σ* Dev**
KLyo Average	AFS-MC	**0.37**%	**10.71**%
	AFS-prev	**0.37**%	10.80%
Kal & KAL_D Average	AFS-MC	**0.19**%	8.43%
	AFS-prev	0.33%	**6.65**%

AFS-MC: AFS-MetaCache, AFS-prev: previous AFS pipeline, *Σ* FP: Sum of all false positive read classifications, *Σ* Dev: Sum of absolute deviations to the given meat composition

### Detection of microbiota

**Fig. 4:**
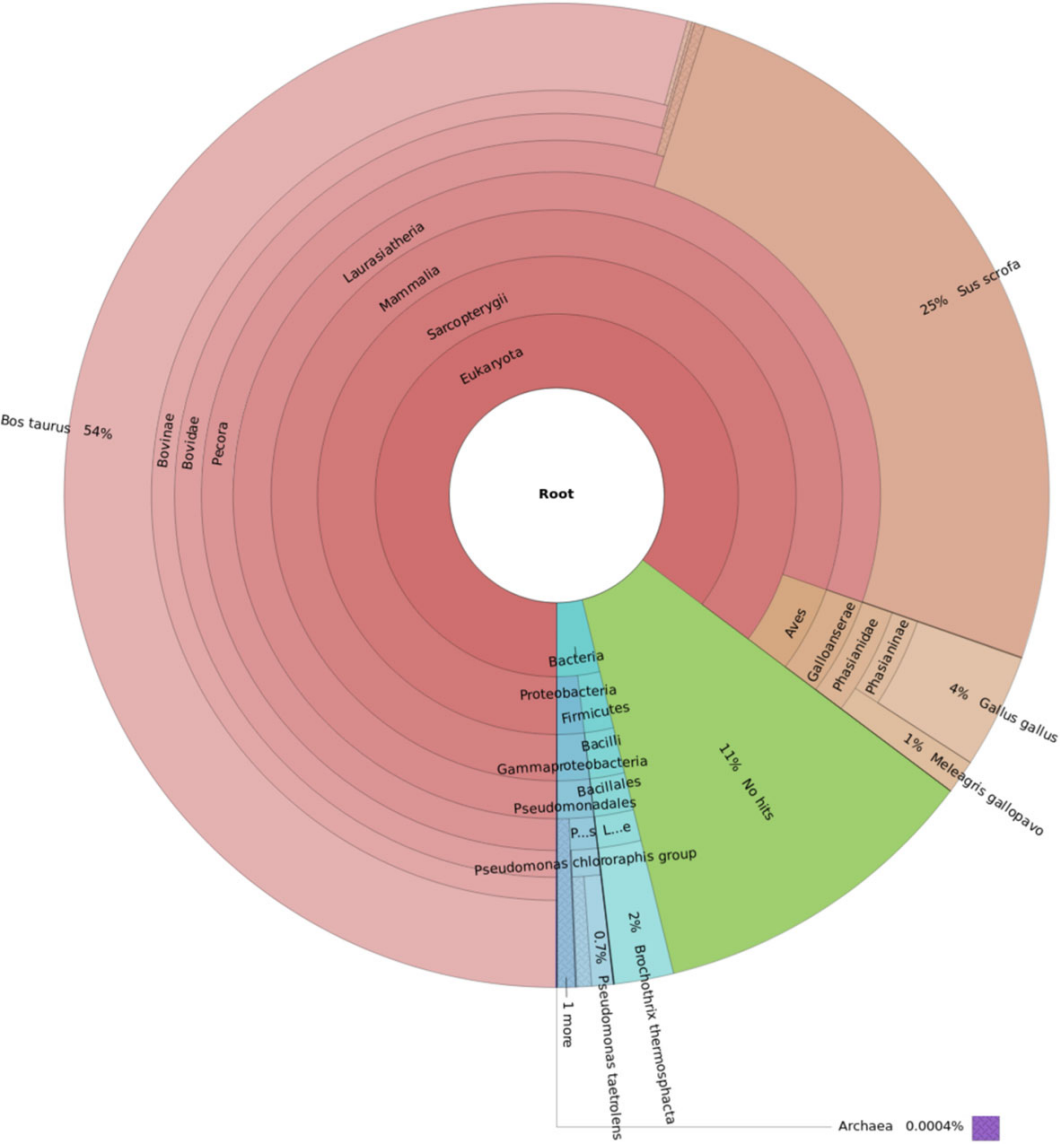
Visualization of the AFS-MetaCache results using Krona [31] for the dataset KLyo_C using the AFS31RS90 reference data set

A major strength of next generation sequencing when applied to foodstuffs, is its theoretically infinite range of species that can be detected. We therefore analyzed the microbiota detected by AFS-MetaCache and MetaCacheSpark in more detail. A visualization of the AFS-MetaCache results using Krona [31] for the dataset KLyo_C using the AFS31RS90 reference data set is shown in Fig. 4. The results of Kraken2 and Bracken agree on the most prominent bacteria as shown in Table 11. The detected bacterial genera Brochothrix, Pseudomonas, and Psychrobacter are well known representatives in foodstuffs. In some sausages a very high amount of the species Brochothrix thermosphacta and even the corresponding Brochothrix phage BL3 could be found, possibly indicating meat spoilage. Furthermore, in several cases a significant amount of Actinoalloteichus was initially detected which has no known relation to foodstuff. However, after application of the coverage filter these matches could be detected as false positives and were removed.

**Table 11:** Detected bacteria in dataset KLyo_C using reference dataset AFS31RS90

**Genus**	**AFS-MetaCache**	**Kraken2**	**Kraken2+Bracken**
Brochothrix	1.94%	1.94%	1.98%
Pseudomonas	1.23%	1.73%	1.92%
Psychrobacter	0.59%	1.43%	1.45%

Genera with less 500 than hits (<0.1*%* of the dataset) are omitted

**Fig. 5:**
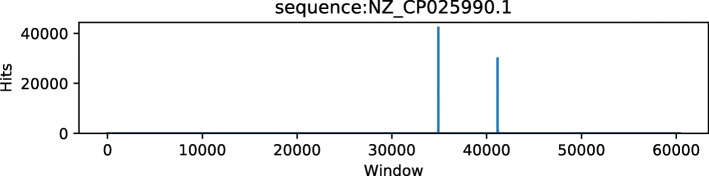
Genome coverage of Actinoalloteichus for the dataset KLyo_C. The sparse coverage is an indicator for false positives

**Fig. 6:**
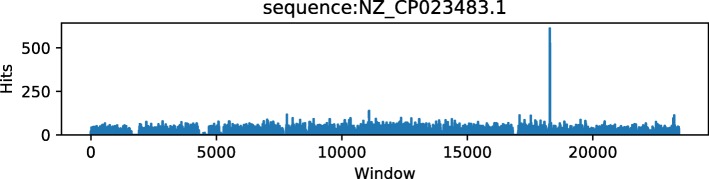
Genome coverage of Brochothrix thermosphacta for the dataset KLyo_C. The even coverage is an indicator for true positives

Figures 5 and 6 show the corresponding genome coverage diagrams for Actinoalloteichus and Brochothrix thermosphacta for the KLyo_C read dataset. The highly uneven genome coverage of Actinoalloteichus is taken as an indicator by AFS-MetaCache for a false-positive species identification. The Brochothrix genome is evenly covered by reads and is thus classified as a true positive.

## Discussion

The determination and quantification of food ingredients is an important issue in official food control [1]. Furthermore, microbiological contamination or the presence of non-declared allergenic food components establishes the need for a broad-scale screening method that allows for precise determination and quantification of ingredients ideally spanning all kingdoms of life including plants, animals, fungi, and bacteria. DNA-based methods like quantitative real-time PCR are established technologies for analyzing foodstuff. However, they have the drawback of being limited to a set of target species within a single assay that need to be defined beforehand. The usage of next-generation sequencing of total genomic DNA from biological samples followed by bioinformatics analyses based on comparisons to available reference genomes can overcome this limitation. Our previous alignment-based AFS-pipeline was found suitable to screen for species in processed food samples [5, 6]. However, the utilized algorithms put limitations on the number species to be screened and on the computational throughput.

Here, we have presented AFS-MetaCache and MetaCacheSpark as new computational methods for the efficient detection and quantification of species composition in food samples from sequencing reads. Being based on an alignment-free exact *k*-mer matching approach, we gain significant speed compared to our previous alignment-based AFS method at the expense of a higher memory consumption for constructing and querying reference genome databases. We apply an intelligent subsampling technique based on minhashing within local windows to reduce the database size. Further reductions of peak memory consumption can be achieved by the introduced partitioning schemes either for single workstations (AFS-MetaCache) or for big data clusters (MetaCacheSpark) at the expense of query speed. Applications of our previous alignment-based AFS pipeline have been limited to around ten complex genomes. With AFS-MetaCache we are able to significantly extend this limit, which is of high importance since the amount of available reference genomes continues to grow rapidly [32, 33]. Thus, our results are particularly encouraging since AFS-MetaCache and MetaCacheSpark are fastest in terms of database construction times. Corresponding peak memory consumption is competitive and can be even further reduced by the partitioned version of AFS-MetaCache on a single workstation or by using MetaCacheSpark on a big data cluster.

While AFS-Metacache can achieve higher query speed than MetaCacheSpark, it takes some manual setup for the partitioned version. MetaCacheSpark on the other hand allows for faster database creation and can easily be deployed on existing Spark infrastructure, while being faster than the partitioned version of AFS-Metacache. Spark, while being fault tolerant, also enables to use a cluster of lower powered computers than we used for our benchmarks.

Within this study we have applied our approach on a broad set of reference samples, containing admixtures of a set of food relevant ingredients (chicken, turkey, pork, beef, horse, sheep). The results demonstrate that our approach is able to reliably detect the components even at the 0.5% level. The comparison to the established metagenomics tools Kraken2, CLARK, and Kraken2+Bracken shows that AFS-MetaCache and MetaCacheSpark are superior in terms of false positive (FP) rates. In particular for pairs of closely related genomes AFS-MetaCache can achieve almost an order-of-magnitude lower FP-rates. These results demonstrate that our classification approach based on counting *k*-mer matches within small windows is effective compared to simply counting *k*-mer matches over an entire genome (as used by CLARK and Kraken) and to an alignment-based approach (as used by the our previous AFS pipeline). Our results also show that AFS-MetaCache achieves the lowest sum of absolute deviations to the included food ingredients. As different types of tissue can contain different concentrations of DNA (matrix effect), deviations could possibly be further reduced by a subsequent normalization procedure that takes tissue ratios into account.

Applications of AFS-MetaCache and MetaCacheSpark are not limited to the study of foodstuff but can be used to analyze high throughput sequencing datasets of metagenomic DNA from other complex biological samples as well, including diverse environmental materials, in-vitro cell cultures, and biopharmaca.

## Conclusion

We have presented a fast screening and quantification method together with two corresponding publicly available implementations (AFS-MetaCache and MetaCacheSpark) for whole genome shotgun sequencing-based biosurveillance applications such as food testing. By relying on a big data approach our approach can scale efficiently towards large-scale collections of complex eukaryotic and bacterial reference genomes making both tools suitable for broad-scale metagenomic screening applications.

## Availability and requirements

Project name: AFS-MetaCache Project home page: https://muellan.github.io/metacache/afs.htmlOperating system(s): Linux Programming language: C++ Other requirements: gccLicense: GPL-3Any restrictions to use by non-academics: according to license

Project name: MetaCacheSpark Project home page: https://github.com/jmabuin/MetaCacheSparkhttps://github.com/jmabuin/MetaCacheSparkOperating system(s): Linux Programming language: Java and C++ Other requirements: Openjdk, gcc, Spark, HadoopLicense: GPL-3Any restrictions to use by non-academics: according to license

## Abbreviations

AFS: All-Food-Sequencing
ddPCR: Droplet digital real-time polymerase chain reaction
HDFS: Hadoop distributed file system
LCA: Lowest common ancestor
LSH: Locality sensitive hashing
qPCR: Quantitative real-time polymerase chain reaction
RDD: Resilient distributed datasets
*Σ*FP: Sum of all false positive read classifications
*Σ* Dev: Sum of absolute deviations
